# Ocean Literacy and Surfing: Understanding How Interactions in Coastal Ecosystems Inform Blue Space User’s Awareness of the Ocean

**DOI:** 10.3390/ijerph18115819

**Published:** 2021-05-28

**Authors:** Natalie Fox, Jamie Marshall, Dorothy Jane Dankel

**Affiliations:** 1 Faculty of Science and Engineering, Anglia Ruskin University, East Road, Cambridge CB1 1PT, UK; nvf100@student.aru.ac.uk; 2 School of Applied Sciences, Edinburgh Napier University, 9 Sighthill Court, Edinburgh EH11 4BN, UK; james.marshall@napier.ac.uk; 3 Department of Biological Sciences, University of Bergen, P.O. Box 7800, 5020 Bergen, Norway

**Keywords:** ocean sustainability, human geography, oceans and human health, ocean literacy, blue space activity, marine social-ecological systems, surfing

## Abstract

Intergovernmental policy is targeting public ocean literacy to help achieve the societal changes needed to reach a sustainable ocean agenda within a 10-year timeframe. To create a culture of care for the ocean, which is under threat from Anthropocentric pressures, informed ocean citizens are central to upholding meaningful actions and best practices. This research focuses on recreational ocean users, specifically surfers and how their blue space activities may inform understanding of ocean processes and human-ocean interconnections. The Ocean Literacy Principles were used to assess ocean awareness through surfing interactions. An online survey questionnaire was completed by 249 participants and reduced to a smaller sample focus group. Qualitative and quantitative data were triangulated to develop further understanding of surfer experiences, using the social-ecological systems framework to model surfing outcomes. The results found that surfers indeed receive ocean literacy benefits, specifically three out of the seven Ocean Literacy Principles and that ocean literacy is a direct benefit many surfers in the sample group receive. By identifying synergies between the Ocean Literacy Principles, variables within coastal ecosystems and user (surfer) interactions, this research offers novel insight into opportunities for integrating ocean sustainability strategies through blue space activity mechanisms and coastal community engagement.

## 1. Introduction

The Anthropocene has imposed great pressure on ocean ecosystems. With climate change, biodiversity loss and marine plastic pollution [1,2,3,4] leading to 59% of the ocean experiencing increasing impact [5], ocean sustainability strategies are beginning to be embedded within international policy [6]. Management of the global ocean and its resources are key targets of the United Nation’s Sustainable Development Goals and specific to Goal 14: Life Below Water [7], yet there are many knowledge gaps when it comes to the global ocean, making mismanagement and emergent stressors rife. The United Nations (UN) is addressing this aspect through a multi-scale and transdisciplinary effort to collect and share Ocean Science during the next 10 years, with ocean literacy identified as playing a key role in tackling social challenges within the Decade of Ocean Science (2021–2030) [8].

Ocean literacy is defined as “an understanding of the ocean׳s influence on us—and our influence on the ocean” which, according to recent studies is “fundamental to living and acting sustainably” [9]. While the concept of ocean literacy began in 2002 as a decentralised and collaborative effort within education in the United States to embed Ocean Science into classroom learning [10], it has now been adapted by the Intergovernmental Oceanographic Commission (IOC) of The United Nations Educational, Scientific, and Cultural Organisation (UNESCO) to reach a much wider audience.

The Ocean Literacy Framework is made up of seven principles and 24 concepts and during the last 15 years has grown to be acknowledged as an integral part of marine citizenship [11]. A global platform was launched in 2015, with “Ocean Literacy for all” providing “a global strategy to raise the awareness for the conservation, restoration, and sustainable use of our ocean” [12]. The latest Ocean Decade report states: “improved ocean literacy allows people across the globe to understand the significance of the ocean on their well-being” and indicates how increased ocean literacy will benefit humanity [13]. Although this extensive ocean literacy strategy has been developed to coordinate collective action, there is little guidance or evidence about whether wider ocean literacy efforts are of benefit to the ocean and ocean communities. The fact is, these themes are inter-disciplinary, constantly evolving, and particularly novel on the international governance stage, with the human-ocean relationship extremely complex and interconnected [14].

Emerging research through the European-funded Horizon 2020 Seas Oceans and Public Health in Europe Project (SOPHIE) studies these interconnections in depth and provide resources which link the conservation of blue spaces to be necessary to manage, maintain and increase the health and well-being of humans [15,16]. The SOPHIE project has been paramount in defining future oceans and human health research priorities through a Strategic Research Agenda (SRA), which includes helping citizens improve ocean literacy. SOPHIE’s SRA 2 (blue spaces, tourism, and well-being) reports that “coastal tourism or living are likely to be the main ways in which the public knowingly interacts with the ocean” [17]. However, not everyone sees the ocean in the same way. To understand better the processes and problems surrounding the human–ocean relationship, approaches to engage a wider diversity of blue space users and insight into groups with different “perspectives, interrelationships, and dependencies” is suggested [18].

In addition, the inter-disciplinary research area of communities and blue spaces (particularly oceans and seas) is currently limited. International studies about the outdoors, environmental literacy, and human well-being, in comparison have reached a coalescence with a consensus that green spaces are good for health [19,20] and understanding the health benefits of nature helps encourage pro-environmental behaviour [21]. Despite opportunities to explore similar mechanisms within blue spaces and society, there appears to be a lack of (longitudinal and natural experimental) evidence about such linkages [22].

This research introduces a case study to explore ocean literacy mechanisms within recreational ocean activities and user groups in more depth. It considers how ocean literacy might be ingrained into human–ocean interactions with specific reference to surfing.

Surfing takes place in the nearshore, coastal zone and can be a completely solo endeavour, with minimal equipment needed (a surfboard and wetsuit in colder temperatures) [23]. Although its origins lie in an indigenous practice performed by Polynesians and Peruvians [24], it now equates to a global industry worth “between 70 and 130 billion US dollars” with an estimated population of 20 to 35 million surfers [25]. Surf specific waves represent a transient, dynamic, and aquatic location known as a surf break [26]. This surf space [27] is also defined as a surf ecosystem by Lewin and Schaefer [28] and where the environmental and social interactions exist in surfing.

According to Borne, surf culture “bears a striking resemblance to the concept of sustainability; creating an enticing marriage between the two” [29] (p. 224). Lazarow and Olive [30] consider surfing to be a system, a definition that correlates with researchers utilising the systems frameworks in sustainable development and surf tourism-based studies. Arroyo et al. [31,32] merge surf break protection in Mexico with contemporary sustainability studies by zoning in on human and surf ecosystem connections. By incorporating adaptive management techniques, they add to the conversation around surfers playing the role of active marine stakeholders. An important point also included in the work of Martin and Assenov [33], Larson et al. [34], Scheske et al. [35], and Atkin et al. [36].

Although there is an absence of any data about the ocean literacy of surfers within scientific literature, Ingersoll [37] testifies that oceanic literacy is intertwined within surfer’s ancestral knowledge and is an essential part of indigenous Hawaiians’ past, present and future. Mills and Bahfen [38] challenge the narrow definition of literacy in terms of writing, spelling, and grammar. They portray surfers as a group who develop the ability to read and ride ocean waves which mirrors a unique aspect of integrated learning, highlighted as important cultural knowledge. Reineman [39] refers to this same aspect as wave knowledge and an essential component for an advancing surfer. It is responsible and required for surfers to progress towards more critical waves, which break in more challenging surfing habitats such as beaches, reefs, and rocky points [40]. Previous studies have indicated that surfers not only receive physical [41], mental [42], emotional [43], and inter-personal [44] benefits from surfing waves but aware and knowledgeable of the ocean system is integral to surviving and progressing as a surfer [39].

In exploring the convergence zone of surfers and their awareness of the ocean, the hypothesis is that surfing produces ocean literacy (which its participants utilise) and is therefore, a potential mechanism for increased ocean literacy. By sampling a group of the surfing population, based predominantly in Europe, primary data was collected to reveal surfer feedback on ocean literacy principles. This research considers how the dynamic, fluctuating and highly varied surfing system might directly relate to ocean literacy learning pathways within coastal communities and is an opportunity to develop further ocean sustainability social awareness and engagement strategies.

## 2. Methods

A mixed-methods approach was used to explore the hypothesis that surfers are ocean literate due to regular exposure to coastal and ocean system variables and their ability to navigate surf breaks (to receive benefits and avoid risks) naturally equips them with ocean literacy. A collection of qualitative and quantitative surfer data was collected to balance resource user feedback (surfer opinions, thoughts, and experiences) with statistical data from a sample of the surfing population. The main objective of the study was to ascertain whether Ocean Literacy Principles are statements surfers agree with and whether surfer variables, such as how often they surf, or their ability, might impact their answers on the Ocean Literacy (OL) Principles.

The research followed a sequential explanatory design, starting with quantitative data collection and analysis and leading onto more in-depth qualitative data collection based on individual (surfer) perspectives [45] (p. 211). The qualitative data results serve to elucidate the statistical data and add a deeper understanding of surfer experiences. Delving into this more subjective aspect enables research to build a clearer picture of exactly how surfers see themselves interacting with the sea, which Britton and Foley [46] describe as “shaping a sense of being and belonging” and important to establish in this novel area.

As the research project took place during the first COVID-19 lockdown procedures (May 2020), in situ research collection at the beach or in person was not possible. Many countries implemented a ban on surfing, therefore due to social distancing and quarantine limitations, an anonymised online survey was produced. Participants of the questionnaire were given the option to sign up to a focus group at the end of the survey by entering their email address and from this a small group were randomly selected and contacted. Demographics were deliberately left out of the first survey to focus on surfer variables (and avoid participant drop off from too many questions). However, a demographic survey was released to ensure that a range of ages, genders, nationalities, and ethnicities across surfers in Europe were represented. Informed consent was obtained from all subjects involved in the study, with participants filling in consent forms for the survey questionnaire and qualitative data collection.

Ethical approval was received by the GSI School Research Ethics Panel (SREP) for a period of 1 year from 21 May 2020 under the terms of Anglia Ruskin University’s Research Ethics Policy. Reference number: GSISREP-1920-002.

### 2.1. Survey Questionnaire

A total of 249 participants completed the surfing survey which was released on 5 June 2020 via the Online Surveys website through Anglia Ruskin University [47] and distributed for 1 month. Social media platforms (Facebook, Instagram, Twitter) and a newsletter connected to the researcher’s website [48] were used to reach participants, as well as contacting surf brands (Quiksilver, Roxy, Volcom, Finisterre), media (Surf Girl and Carve Magazine), Non Government Organisations (Changing Tides Foundation, Surfers Against Sewage reps community, and Surf Rider Europe), professional organisations (surf schools through Surfing England database) and high profile surfers (Belinda Baggs; Patagonia Australia ambassador) in an attempt to recruit further participants. Survey participants were asked to share with friends and their own surfing communities to create a snowball effect [49]. In statistical analysis, an ideal (minimum) sample size of 384 participants would be used to have a confidence interval of 5% and a confidence level of 95% to represent the (average estimated) mean population of 27,500,000 surfers. Instead, a sample size of 249 reduces the confidence interval to 6.21% and a confidence level of 93.79%. The sample is considered sufficient as statistical researchers work with a confidence level of 90% and above [50] (p. 168).

There were 12 questions, with the first 4 determining surfer variables based on a diving study looking at the motivation, experience, frequency, and location of divers in relation to environmental engagement [51]. The surfer profile was adapted to quantify experience (how long have you been surfing?), frequency (How often do you go surfing?), localisation (Do you have a surf break you prefer to use?), and ability (How would describe your surfing level?). A Likert scale was used as a psychometric tool to collect and categorise answers from less to more across surfer variables and these totals were used to test for independence when cross-tabulating with further data [52].

Questions 5–7 asked about surfing motivation, barriers, and emotional impact of aforementioned barriers. In Question 5 (What are the benefits you receive from surfing?) the answer “connection with the ocean” was used to represent ocean literacy aspects of surfing (awareness, influence, understanding). For question 8 participants were only asked to answer YES or NO to whether they agreed with the following statements (1–7 OL principles) to obtain a dichotomous result. They were not informed of the ocean literacy principles or framework. All statements are correct (according to the OL framework) and participants were encouraged to think about the statements before making their selection because it could not be changed. Question 9 asked what was responsible for their previous answer to understand the origins of their proposed ocean literacy.

The research hypothesis is that surfers in the sample group would be intuitively ocean literate. However, due to the absence of scientific evidence of the connection between surfing and ocean literacy, the research also followed the assumption that the terminology “ocean literate” is not prominently used by surfers (either in general or to describe their knowledge/themselves). Due to its origins within the education sector (rather than surfing mainstream) and connotations literacy has with reading and writing, “ocean literacy” is a term much more likely to be used by scientists, teachers, or academics [53], which, of course, there may be a crossover within the sample group. Questions 9–12 shifted focus to ask about the influence surfing may have had on participant’s appreciation, awareness, and action around ocean ecosystems. Most questions were multi-answer; giving options rather than choices, and “other” answers from the survey were also used in qualitative data analysis (see Appendix A for all questions).

Chi-squared (*χ*^2^) tests were used to assess the statistical significance of the quantitative findings. The surfer variables (experience, frequency, localisation, ability) were tested for independence against the other variables using a *χ*^2^ test for independence to determine whether or not there was a significant association between categorical variables.

For the ocean literacy principles, a *χ*^2^ goodness of fit test was used to test the null hypothesis that surfers are not ocean literate by testing observed frequencies of YES and NO answers against expected frequencies. The *χ*^2^ tests for independence were run on the cross tabulated answers of ocean literacy YES or NO and surfer categorical variables, to determine statistically significant dependence. The null hypothesis was that surfer variables are independent to the answer of YES or NO for each of the 7 Ocean Literacy principles.

### 2.2. Focus Group

A total of 70 participants entered their email addresses to be considered for the online focus group. The focus group approach was used to established rhetoric with participants to collect qualitative data, much like Usher et al. [54], who suggest that research about local surfers be used to inform surf management practices. Further qualitative research to “deepen our understanding of individuals” lifelong experiences of coasts will also help explore the meaning that users attach to “blue space” [55]. Once the survey had closed and the data analysed, 13 participants were chosen at random from the list of emails, contact was established, and the final 6 were chosen according to their availability, location (European time zone), and surfer variables. The ratio of male to female participants was 2:4. The rest of the surfer demographics can be found in Appendix B. An online focus group via Zoom was scheduled for 29 July 2020 via email which included a briefing for the participants on the format (See Appendix C). The session used a semi-structured format which started with a discussion about how “Surfing requires ocean literacy”, a quote by Parsemain in Mills and Bahfren [38]. The researcher guided the participants to speak about their ocean literacy thoughts, followed by the benefits they receive, barriers to, and risks they encounter through surfing. The session lasted for approximately 1 h and once the focus group recording had been transcribed and uploaded into Nvivo, the data was analysed and codes were assigned by hand. These codes were turned into themes and developed into open-ended questions (See Appendix D for themes).

### 2.3. Online Questions

A written interview comprised of the following 6 open-ended questions.

1. Ocean literacy is defined as “an understanding of the ocean׳s influence on you—and your influence on the ocean”. Would you say that learning to read waves for surfing purposes has made you more ocean literate? If yes, can you give a specific example?
2. Have you noticed small particles of plastic or fishing gear in the surf breaks that you frequent, and as more evidence of microplastic pollution is scientifically published are you concerned about ingesting microplastics or microplastics damaging your local surf break/ocean ecosystem? Would it stop you from surfing?
3. In terms of surfing, do you use it as a therapeutic benefit to help maintain good mental health? If so, can you describe how?
4. Can you explain any negative emotional impacts surfing in or witnessing plastic pollution in your local surf break triggers?
5. Marine microplastics are now notoriously ubiquitous and widespread. How do you think we can solve the (micro)plastic pollution crisis? Would humanity becoming more ocean literate and more systems literate be part of it? (Systems thinking is a holistic approach to analysis that focuses on the way a system’s constituent parts interrelate and how systems work over time and within the context of larger systems. Systems literate is shifting from analytical thinking to contextual thinking and emphasising relationship-based processes such as cooperation and consensus).
6. As a surfer would you say you employ certain aspects of systems thinking to receive benefits such as: physical exertion, mental clarity, connection to the ocean and fun and avoid risks such as: bad conditions, overcrowding, injury?

These were sent out to the same participants via email to obtain specific qualitative answers, with data later coded to find evidence of ocean literacy principles, clarify quantitative data observations, as well as to track any emergent themes about the wider concept of marine conservation. Having spent time discussing ocean literacy in the focus group as a primer, these questions went deeper into connections between surfing and ocean literacy. After all qualitative data was collected, evidence of the variables was found through reflective thematic analysis. To find evidence of the ocean literacy principles, the interview data was subjected to a text query using key words from each ocean literacy principle.

### 2.4. Social Ecological Systems (SES) Framework

The Social Ecological Systems (SES) framework was used as an overarching conceptual methodology while conducting the research [56]. Within the sustainability field, at least 10 frameworks have been identified for analysing anthropogenic and environmental system interaction. The SES framework was chosen specifically, due to its use in previous studies (sustainability and surfing methodology) and because it is the only one that treats interconnected social and ecological elements almost equally, providing an appropriate frame for exploring variables [57]. Triangulation of quantitative and qualitative surfer data was used to confirm validity and reliability of the results, zooming out to view findings through the SES framework lens [58]. Finally, a version of the SES model was developed to display key ocean literacy interactions and outcomes within the surfing system.

## 3. Results

### 3.1. Quantitative Results

#### 3.1.1. Surfer Experience, Frequency, Localisation, and Ability

The results from the surfer variables showed there were higher frequencies of certain “categories” of surfers, including: 52.6% surfing for more than 10 years; 46.2% surfing 1–5 times a week; 43.4% moving between a few different spots in their local area; and 45.2% of intermediate ability. The results from the statistical tests found that the categories do not fit a uniform distribution, as shown in Table 1.

#### 3.1.2. Connection to the Ocean

The survey asked participants to name the main driver(s) behind why they surf, the origins of their ocean awareness, and to scale how much they valued the ocean. Descriptive statistics showed a tendency towards a high understanding, awareness, and appreciation of ocean ecosystems. “Ocean literacy” was substituted for “connection to the ocean” to represent enjoying the relational aspects of surfing. The highest number (91.2%) of participants choose “connection to the ocean” as a motivating factor for surfing, with 77.4% of participants attributing surfing to their understanding of the ocean (also the highest and almost twice as likely as school or University). 55.8% of participants highly value ecosystems due to surfing (ranked 1st). In terms of threats to surfing ecosystems 72.7% of surfers had become aware of plastic pollution through surfing in multiple ways and 95.5% said they would take action by cleaning plastic off the beach (both the highest tendencies). 59.2% of participants said they would sign up to a citizen science programme and 42.2% to a focus group, to help ocean conservation. However, only 71 people entered their email address to join the focus group, showing that given the opportunity only 28.5% of participants would actually engage.

#### 3.1.3. Ocean Literacy Principles

Table 2 collates the Ocean Literacy Principles answers into rank order starting with the highest frequency. In total, all statements had an <81% agreement. Although there were discrepancies due to some participants answering twice (or not at all), the results indicate that a high number of surfers in the sample group agree with each of the ocean literacy principles. All of the survey participants agreed with Ocean Literacy Principle 5: The ocean supports a great diversity of life and ecosystems.

#### 3.1.4. Variables and Ocean Literacy Principles

The surfer variable data were cross tabulated with the Ocean Literacy Principle answers to test for dependency. The null hypothesis was that no relationships exist between the categorical variables (the hypothesis is that the categorical variables are dependent). The statistical analysis found no correlation of significance except between Frequency and Ocean Literacy Principles 2 and 6. For these, the *p*-value was <0.05, and therefore, we can accept there is a relationship between the frequency variable and certain ocean understanding. The results show that how often surfers go surfing is significant with regards to their understanding of “the ocean shaping the features of Earth” (OL Principle 2) and “the ocean and humans being interconnected” (OL Principle 6). (See Appendix E for contingency table).

### 3.2. Qualitative Results

The qualitative data shows how there are many variations in ocean literacy outcomes. Despite similarities, surfers experience differences throughout their individual surfing interactions with the variables helping explain and categorise these differences.

Experience is key to being able to read waves, with Participant E embracing the learning process.

*“* *As I’m still a beginner/improver surfer, I’m not at the point where my reading of waves necessarily means I catch more of them, but what I love is the process of trying to understand each individual break that I visit.”*

Participant C noted how having time out from surfing brought about more appreciation for it, which relates to frequency.

*“* *I think there’s definitely such a thing as a surfing withdrawal symptoms, if you haven’t surfed in a while. If you’ve been stuck on a job or in a city, and then you get back to the sea, you’re just so thrilled to be in the water.”*

Results showed that surfing benefits vary due to surfing manoeuvres and conditions, but are still positive, if, such as Participant C, the surfer has a level of ability.

*“If I’ve had a really good surf, like a stand-up barrel or something, I’m just, I’m over the moon. It’s like a hundred percent.”*

Having a local beach means the privilege of access to physical and emotional benefits of surfing, which also links to localisation.

*“We’d just done a really long journey and got straight out to the car and went for a quick surf. It was really sloppy conditions. It was just a bit of fun and a bit of exercise.... but it helped to stretch and de-stress.”* (Participant A)

Motivation to surf and the positive outcomes (in reference to connection) showed up in the qualitative data, including: experiential learning; *“It helps educate me with life lessons and allows me to demonstrate that I am capable of overcoming overwhelming problems”*; passion; *“Following a passion for surfing has inspired me to learn and raise my awareness about the interconnections of life and our environment”*; and collaboration; *“There’s a sharing part of the sport that make us belong to a community. Communities evolve together by learning, teaching, sharing, experiencing.”* (Participant F).

Participant D commented on the interactive elements. *“When you’re surfing, you’re part of the ocean, Surfing and its effects are less...static. It not a gentle soak, its participation in the ocean. Participation in its energy and alive-ness.”*

There were a range of barriers mentioned by participants, in addition to conditions and personal reasons (highest answers in the quantitative data) that included: “sharks”; “financial pressures”; “proximity”; “childcare”; and “gender-based abuse”. One participant elaborated further. *“As a woman, it can be intimidating to surf in crowds with men without a friend so this can sometimes hinder whether I get out or not”* (Participant ID: 607219-607210-61306670).

Participants mentioned marine plastic pollution as the most concerning ocean sustainability issue, as opposed to climate change, biodiversity loss and de-coastalisation. They responded with highly emotional and emphatic data in the qualitative analysis.

*“It is sad and frustrating when you leave a surf having collected crisp packets, tampon applicators, and fishing gear on your way out. It deeply affects me when I see the beach covered in plastics. My heart breaks for the creatures of the world.”* (Participant E).

While all were aware of plastic pollution in the marine environment through surfing, when asked whether plastic pollution would actually stop them surfing the answers ranged.

- Participant C *“No. (it would not stop me from surfing)”*
- Participant D *“Yes, not for safety though, just for sadness.”*
- Participant E *“It would if the water was full of it, like it has been in some countries I’ve visited.”*

The “other” answers in the questionnaire showed how surfing could provide both the interaction and the outcome for knowing about these issues.

*“I was aware of plastic pollution before surfing, however it became a more important issue for me through surfing.”*

(Interaction) (Participant ID: 607219-607210-61170018)

*“Having an interest in the ocean for surfing, encourages me to learn more about [plastic pollution] by watching documentaries etc.”*

(Outcome) (Participant ID: 607219-607210-61866951)

The qualitative data showed evidence that surfers employ systems thinking while surfing, but perhaps without knowing it. *“I wouldn’t say that I consciously employ aspects of systems thinking, but can see on reflection that I do”* (Participant E).

Participant B explained how their perspective changes once in the sea.

*“When I’m thinking about going for a surf I think about the conditions and if the waves are going to be good, but once I’m in the water, I notice how clear it is, the temperature, if there’s fish and wildlife.”*

Participant D inserted themselves into a dynamic system, noticing how the benefits they receive rely on the conditions working in their favour.

*“Everything is deeply interrelated. Understanding all the cogs rely on each other is a big part of surfing. Whether that’s conditions that need to come together for a wave to work (tide, wind, swell, etc.) or mental (I have to get cold, or walk a long way to a break, but I know I’ll benefit from it)”.*

All participants confirmed that surfing helped increase their awareness and understanding of the ocean, including participant B.

*“Surfing has definitely made me more ocean literate. Understanding how waves work helps me to choose the right wave (most of the time!), which helps me receive the most benefit from surfing, physically, and mentally.”*

This happens for another, with an added sense of mindfulness.

*“Reading waves slows me down, makes me more mindful of my surroundings and of nature, and takes me out of all of the other stuff in my head.”* (Participant E).

While the qualitative data failed to produce any evidence of Ocean Literacy Principles 1, 2, 4, and 7, the other three Principles were acknowledged by participants. Participant C admitted using intuition regarding the weather and talked about how sensory perception helped to predict surf conditions in different locations. *“When I was a teenage surfer in Cape Town, South Africa I could tell what the conditions were like when I woke up in the morning. Now after living in Croyde, United Kingdom for the past 5 years, I can do the same thing. I think human beings can feel atmospheric pressure, which relates to winds. Temperatures are also relevant to pressure.”* (OL Principle 3: *The ocean is a major influence on the weather and climate*).

Exposure to marine pollution in surfing environments has made participants think about and take action to protect ocean ecosystems., for example participant C. *“I have picked out a few pieces of plastic from the ocean when I have been surfing and considering how much trash we collect on beach cleans, I think ocean ecosystems are already being damaged by microplastics and trash.”* (OL Principle 5: *The ocean supports a great diversity of life and ecosystems*).

One participant commented on how being in sea water provided therapeutic benefits. *“I have a more emotional connection to the water since surfing, I am more aware on its effects on my mental well-being.”* (Participant D) (OL Principle 6: *The ocean and humans are inextricably interconnected*).

**Figure 1:**
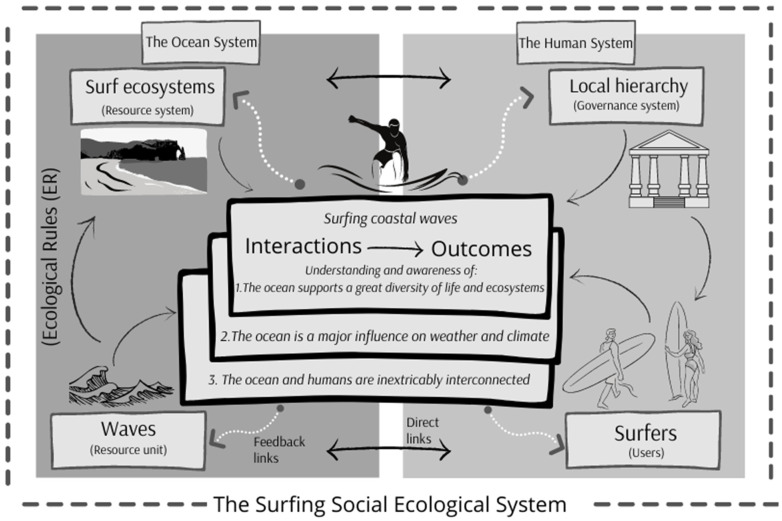
The Surfing Social Ecological System model is adapted from McGinnis and Ostrom [59] and demonstrates how the human (social) and ocean (ecological) systems provide opportunities for interactions between surfers (users) and waves (resource units), producing ocean literacy understanding and awareness.

The mixed-methods approach culminated with a cross section of results from the survey and interviews regarding evidence of ocean literacy. The findings confirmed that the Ocean Literacy Principles from the surfer sample population were most familiar with were Principles 3, 5, and 6 (with an absence of evidence of Principles 1, 2, 4, and 7 in the qualitative data). These findings were built into a surfing SES model as seen in Figure 1.

## 4. Discussion

### 4.1. Ocean Literacy Learning Pathways through Surfing

This study serves to provide a sound basis for the argument that ocean literacy is an intrinsic benefit received by surfers, through the coastal-based recreational activity of surfing. Concluding whether surfing is a reliable and consistent mechanism for ocean literacy, however, is still open for debate. A self-assessment protocol was used to determine ocean literacy levels of surfers, which has its limitations and does not necessarily equate to actual, observed levels of ocean literacy. While the quantitative data results imply that surfing participants are knowledgeable across all Ocean Literacy Principles (more than 80% in agreement with all 7 principles), the qualitative results reveal gaps with an absence of ocean literacy principles 1, 2, 4, and 7, in the analysis.

Therefore, to measure more accurately in future research, it would be worth drawing on systematic methodology devised by Brennan et al. [14]. They amalgamated various definitions of literacy to create learning dimensions for the ResponSEAble project study, including awareness, knowledge, attitude, communication, behaviour, and activism and once participants had been exposed to ocean literacy learning, researchers quantified changes that took place within these dimensions. While Brennan et al.’s tool is designed to improve ocean literacy through systems thinking, work by Fauvile et al. to develop the International Ocean Literacy survey is a more extensive framework that could be adapted to assess surfer ocean literacy [60]. Incorporating a Rasch model for measurement could help quantify surfer ability to read ocean variables rather than the Likert scale methodology which tends to reflect personal attitude, and research continues to determine the most accurate, appropriate and specific methodology to reflect ocean literacy learning [61].

Research by Reineman [39] concludes that “incorporating surfers wave knowledge could result in more equitable decision-making regarding coastal management” and while surfers exhibit extensive attachment to their frequented surf breaks [62], there is no mention of ocean literacy. The results of this study support the argument that not only do surfers possess wave knowledge but knowledge of ocean ecosystems. Furthermore, this deep sense of connection to the ocean is an equivalent of ocean literacy. With immense effort going into the unilateral ocean literacy portal and platform, this study highlights the inter-disciplinary nature of surfing and identifies a potential correlation to the ocean literacy framework. Strengthening knowledge of this within surfing communities, industry and culture could encourage surfers to be more ocean literate and fill knowledge gaps around the principles. Increasing the network between ocean literate surfers and policy makers could support a better understanding of surfing habitats. Communication between both is vital for management of ecosystems services and a shared language is needed for knowledgeable stakeholders to help inform policy and decision making. Rather than creating a new framework, such as “wave knowledge” Reineman [39], it would be best to use systems thinking and strengthen the networks, interactions and relationships that already exist around ocean literacy, saving time and resources in the process. For example, focus group Participant D noted. *“Ocean literacy is a brilliant systems thinking learning tool”* although one that has remained ambiguous in the surfing world. More studies, such as Ferreira et al. [63], who provide an assessment of a non-formal ocean literacy research project could help contribute to understanding how ocean literacy fits into the international agenda for sustainable development and how goals are being accomplished at the local level.

However, focus group Participant A reminds us that *“Ocean literacy is a privilege, extended only to the very few who get to live by the water, or can afford to visit it.”* which is also the case for surfing. Privilege, access, and inequity are themes beyond the scope of this study but are what De Bell et al. [64] address as more cautionary aspects of blue space. These themes are most definitely recommended for further investigation. Exploring geopolitics, gender, ethnicity, and poverty within the context of surfing ties into existing studies of surfer protests in Lima [65], exploitation within the surfing mecca of Indonesia [66], and a study of surf therapy in post-conflict Liberia [67]. The results of this research indicate that barriers in surfing are also rife and there is a need to explore more complex and urgent social issues, while managing to maintain an ocean sustainability agenda, in other words “ocean justice” [68].

When considering social aspects of surfing in terms of health benefits, there is literature concerning the therapeutic benefits of blue space [69,70]; yet surfing studies are relatively novel [71,72]. These results support how surfing might challenge complexities around personal identity and social/cultural norms and in particular, hydrophilic place-based “belonging” narratives [73,74]. This study also supports the argument that there needs to be emphasis on updating and decolonising ocean governance perspectives, while listening to the most marginalised voices within coastal communities [75]. Working with surf communities across developed and developing counties in this way could provide valuable insight into how best to develop standardisation of ocean literacy across the nuance of social backgrounds. At the same time as encouraging inclusive and diverse community engagement and maintaining rigorous best ocean practices [76].

Surfing, in this sense, is a key case study on how humans interact with the ocean as it “reflects a relationship to practice, place, and community that enables and restricts, but this complexity is difficult to articulate” [73]. Whilst this this research attempts to start to unravel such complexities, it also links with the work of Olive and Wheaton [73], who study how blue spaces are used and experienced to “recognise the ongoing impacts of colonisation, racism, xenophobia, homophobia, and exclusion.” While these issues were not at the forefront of this particular study, barriers such as gender-based abuse and access expressed in the qualitative analysis call for further investigation.

### 4.2. Surfing as Informal Ocean Science and Sustainability Education

The overall results from this study provide extensive evidence that conceptualising surfing as a social-ecological system (SES) is an appropriate framework to explore the inter-disciplinary and interconnecting components of the surfing system. Within the surfing system, evidence of Ocean Literacy Principles shows that surfing is an example of informal education. This outdoor, out of classroom learning that Sacco et al. [77] call “lifelong, life-wide and life-deep” is also evident in the 50% of participants who had been surfing for more than ten years, as well as the 55.8% of participants who highly value ecosystems due to surfing.

Bourne [29] (p. 222) uses the metamorphosis model to communicate processes, transitions, and future pathways of the surfing subculture. However, viewing surfing in this way misses out the necessary ecological factors and emergent pressures that contribute to the surfing system, which could influence and add to the ocean literacy of surfers. Therefore, this research calls on the updated work of McGinnis and Ostrom [58] as a highly useful model to dissect, discern and chart the future of surfing within the wider ocean system and harness opportunities to observe feedback loops of knowledge, action, and behaviours within the surfer user group.

In terms of sustainability, surfing is not exempt from systems archetypes such as “Limits to growth” [78] (p. 27) and “Tragedy of the commons” [79]. In fact, Nazer [80] addresses this in the paper “Tragicomedy of the surfer commons” with the argument that surfers have so far successfully managed their “commons” through cultural norms and rules. However, by providing evidence of the mental and emotional toll environmental issues, such as marine plastic pollution has on surfers, this research argues that much more care and custodianship needs to go into protecting the resource units and systems (waves, surf-breaks, coastal habitats, and the ocean) of surfing or, at the very least, monitoring current states. This is applicable for both blue spaces and blue space users.

The global network of surfers is now united by the inclusion of surfing in the (2020) Olympics [81]. This signifies a tipping point into the mainstream for surfing where its global growth may provide opportunities (leading to more tiers, interactions, outcomes, and feedback), but will inevitably lead to more complexity and therefore more challenges. As the surfing structure grows, it could potentially add pressure to ocean ecosystems. The unique coastal and marine-based spaces surfers visit need to have substantial governance, if they are to carry the weight of frequent and increased use. By fully identifying and developing ocean literacy outcomes, this could be utilised as a leverage point to increase knowledge, change human behaviour, and help create more ocean literate coastal communities [82].

The participants in this research are clearly concerned about the state of local surf ecosystems and the ocean at large, and the coastal zone is where more marine stewards are needed. Surfers have already shown potential to monitor environmental indicators, shifting their role from steward to citizen scientist [83]. Notable scientific surfing projects include the beach bums survey [84], the surfer biome project [85], collecting climate change data via the SMART fin [86], sea temperature and GPS data via surfers [87], marine debris/golfball study [88], and paddle-boarding for microplastics [89]. Significantly, in this study 59.2% of all survey participants said they would contribute to citizen science.

The success of these previous surfing-citizen science studies (in the peer reviewed and published sense), supports the proposal that more scientific research by surfers within surf breaks, however niche, is thoroughly needed to fill knowledge gaps across the inter-disciplinary human ocean system research area. Evidence that surfers would embrace this role is supported by Whyte’s “Saltwater Citizen” theory (whereby surfers take an active, political stance for their surf breaks) [90] and explains the increasing momentum behind surfer-led environmental Non Government Organisations, such as SurfRider Foundation and Surfers Against Sewage. This research area also backs up studies which report increased connectedness to nature (feeling a connection or affinity to nature) influencing attitudes and behaviours that support the sustainable use of natural environments [21].

With surfers expressing concern regarding plastic pollution in this study, the question to ask is to what extend can they be relied on to continue the management of surf breaks? To maintain healthy and functioning surf ecosystems, sustainability principles, and frameworks of sustainable development are needed to outweigh incentive for growth [91] and address the complexities of emergent pressures, such as marine plastic or climate change.

This study echoes Scheske et al. [35], who call on alliances between surf ecosystem users (surfers) and marine conservation groups, along with policy, to address the sustainability challenges that exist in the surf space. There is a delicate biodiversity where coastline meets ocean, yet both are invested stakeholders. With this in mind, there are major opportunities within inter-disciplinary fields of sustainability and marine policy to utilise surfing as a key case study. There is huge scope to engage with surfing stakeholders through OL and SES frameworks. By combining Ocean Decade social strategies with the Olympic world stage there is an opportunity to champion a sustainable future for ocean communities, as well as highly-precious surf ecosystems, home to an abundance of social benefits and ecological resources.

### 4.3. Synergies in Surfing, Ocean Literacy, and Ocean Sustainability

Scientific disciplines have only recently come together to consider the importance of how Earth’s bodies of water (human and ocean) interact [92]. Even the ocean literacy framework includes the concept of known unknowns through “the ocean is largely unexplored” (Principle 7). Yet, surfing’s heritage has cultivated a level of ocean understanding and awareness for hundreds of years. Despite surfing’s current Western-dominant and linear mindset [93], surfers are united by their dependence on reading the dynamic processes of the: ocean, tides, surface waters, currents, and waves. Decoding this knowledge and understanding has been passed down through cultural ties and, though technological advancements may have had some impact, surfing still requires humans to read the weather and the ocean to support desired outcomes [94]. Sustainability and systems thinking teaches that the social and ecological components of this world are not necessarily separate. Instead, they are fully and intricately interconnected and this research starts the conversation around synergies between surfing communities and the ocean system.

This study shows surfers, and potentially other recreational ocean users, use systems thinking at an intuitive level. This is an exciting prospect when considering opportunities to increase public ocean literacy within inter-disciplinary marine science. Also, thinking systemically is key to rebalancing and reframing ocean sustainability on a global scale, with collaboration and joint action at the core.

Ocean literacy could be a vital tool to awaken non-surfing humans to their dynamic relationship with the ocean, inspiring action to not only understand better, but to take on a role of stewardship and help drive societal behaviour change [95,96]. While the findings of this study concentrate on one user group (surfers), further research is suggested to assess if and how ocean literacy is embedded within other ocean recreational activities. Comparison studies (between surfers, divers, swimmers) or the use of control groups (non-surfers) could help determine ocean literacy mechanisms and look deeper into the causal events that occur though exposure to ocean ecosystems.

According to the research embedded in the Seas Oceans and Public Health project, improving ocean literacy levels could also help improve understanding of risks and benefits to health from interacting with the ocean. A recent meta-analysis reflects how multiple marine stakeholders have different priorities, therefore, citizen engagement needs to consider this whilst working systemically and taking a participatory approach [15]. Self-efficacy and emotional involvement have also been identified as pre-cursors to pro-environmental behaviours [97]. This research found considerable mental health (associated with mindfulness) benefits that arise from surfing. It also concurs that consideration of the negative emotional risks to mental health from being exposed to polluted ocean and surf ecosystems is advised. Due to the diversity of variables within the surfing SES, more research into emergent entities within the global surf community is suggested. Research investigating the variations of user subgroups (gender, age, ethnicity, nationality) and choice of craft (longboarders, shortboarders, or bodyboarders) or financial incentive (professional surfers, recreational, tourist, and surf schools) would be a start. Working in collaboration with surfing Non Government Organisations, surf schools, surf brands, and industry giants such as, the World Surf League or the International Surf Association could help future research assess further synergies in oceans and human health, as well as investigating whether pro-environmental actions are triggered by surfing interactions and outcomes. The International Ocean Committee have already shown awareness of this simpatico by featuring famed Brazilian surfer Maya Gabeira in their Brave New World Ocean Decade webinar series. As a spokesperson for the Ocean Decade and an accomplished big wave surfer, Gabeira could indeed represent an ocean literate surfer and be an example to the public of how “healthy oceans foster healthy people” [98].

## 5. Conclusions

This study concludes that surfers receive ocean literacy benefits—specifically understanding and awareness of Ocean Literacy Principles 3, 5, and 6—and that surfing waves within coastal ecosystems is a potential mechanism for ocean literacy. There are many variables within the surfing SES, which produce a variation of interactions and outcomes, yet the results show frequency—how often surfers surf—is statistically significant with regards to surfer ocean literacy levels. Despite extensive data collection, there is not enough scientific evidence to support the theory that the surfing SES contains proven mechanisms for high levels of ocean literacy.

However, this research serves as a foundation on which to build on and argues there is a clear prospect to develop more knowledge about how ocean literacy is produced through the surfing SES. It identifies surfing as an opportunity to extend current ocean literacy learning pathways.

Surfing, rather than simply a sport, lifestyle, or subculture, is an example of the interconnectedness of humans and the ocean, with surfers learning key principles through their surfing exploits, including:

1. the ocean is a major influence on the weather and climate;
2. the ocean supports a great diversity of life and ecosystems;
3. the ocean and humans are inextricably interconnected.

Surfers are untapped data points with levels of ocean literacy that could be useful to ocean science and a prime example of a global ocean community that will soon exceed 35 million participants, who could demonstrate a “culture of care” [15]. There is a need to expand this area of research significantly and especially in the context of the Decade of Ocean Science, when all eyes and ears are on ocean observations. If the human-ocean relationship is to be understood better and the goals of the Ocean Decade achieved (within the timeframe), it is crucial to engage with users of the ocean resource system who are integral to building ocean research multidisciplinary networks. Further research into surfing is a way to support international collaboration while honouring cultural heritage [99] and may lead back to origins and more opportunities for progress in ocean literacy. With increasing emergent pressures from the Anthropocene, the demonstrated convergence zone of surfing and ocean literacy, provides further opportunities for ocean sustainability strategies to invest in fostering social-ecological synergies [100] and work towards creating a sustainable future, not only for coastal and ocean communities, but for the whole of humanity.

## Figures and Tables

**Table 1:** This table displays categorical variables for the first four questions of the survey.

Question		Categorical Variables						χ^2^ Test
1. Surfing experience		A. Under 1 year	B. 1–2 years	C. 2–5 years	D. 5–10 years	**E. Over 10 years**	* Other	** χ^2^ = 191.64
	*n*	12	13	47	43	**131**	3	
	%	4.8	5.2	18.9	17.3	**52.6**	4.8	
2. Surfing frequency		F. Once a year or less	G. Once or twice every 6 months	H. Once or twice a month	**I. 1–5 times a week**	J. Every day	* Other	** χ^2^ = 144.29
	*n*	15	38	59	**115**	14	8	
	%	6	15.3	23.7	**46.2**	5.6	3.2	
3. Surfing localisation		K. Only on holiday or weekends away	L. I will travel to find good surf (condition dependent)	M. I surf my local break, but I also travel extensively to surf	**N. Move between a few different spots in my local area**	O. I have a local break I always go to	* Other	** χ^2^ = 91.58
	*n*	38	29	34	**108**	34	6	
	%	15.3	11.6	13.7	**43.4**	13.7	2.4	
4. Surfing ability		P. Beginner	Q. Improver	**R. Intermediate**	S. Advanced	T. Elite/Professional	* Other	** χ^2^ = 139.06
	*n*	22	55	**112**	56	3	0	
	%	8.9	22.2	**45.2**	22.6	1.2	0	

The mode of each variable is * in bold. Each variable went through a χ^2^ goodness of fit test against an even distribution of expected values (the total divided by number of categories), using the programme R (The R Foundation, Vienna, Austria) m, with the results shown in the final column. * Other answers were not included in χ^2^ tests. ** All *p*-values were less than 0.00001 meaning the null hypothesis (variables are equally distributed) was rejected and the alternative (variables are unequally distributed) was accepted.

**Table 2:** Ocean Literacy Principles as statements that surfers agree or disagree with.

Ocean Literacy Principle	(Observed) YES (*n*)	(Observed) NO (*n*)	Yes (%)
5. The ocean supports a great diversity of life and ecosystems	248	0	100
3. The ocean is a major influence on weather and climate	239	8	* 97
6. The ocean and humans are inextricably interconnected	239	13	* 95
7. The ocean is largely unexplored	239	21	* 92
4. The ocean made the Earth habitable	239	22	* 91
2. The ocean and life in the ocean shape the features of Earth	239	22	* 91
1.The Earth has one big ocean with many features	239	49	* 81

The table shows the Ocean Literacy Principles ranked in relation to total answers for YES or NO. Each answer was tested against expected values—99% YES—using a χ^2^ goodness of fit test. * in the table indicates all principles apart from No.5 showed statistical significance with *p*-values < 0.01.

## Data Availability

The data presented in this study are available on request from the corresponding author. The data are not publicly available due to privacy.

## Appendix A

**Table A1:** Anonymised survey questions.

Questions		Answer
1	How long have you been surfing?	Multiple choice (5 options including other, Likert scale)
2	How often do you go surfing (approximately)?	Multiple choice (5 options including other, Likert scale)
3	Do you have a surf break you prefer to use?	Multiple choice (5 options including other, Likert scale)
4	How would you describe your level of surfing	Multiple choice (5 options including other, Likert scale)
5	What are the benefits you receive from surfing?	Multi-answer (13 options include “open” other)
6	Have you experienced any barriers to stop you receiving benefits from surfing?	Multi-answer (7 options include “open” other)
7	How do you feel if you are unable to go surfing?	Multi-answer (7 options, likert scale)
8	Do you agree with the following statements? The Ocean Literacy Principles	Yes or No
9	What would you say is responsible for your knowledge and understanding of the above statements you answered yes to?	Multi-answer (6 options include “open” other)
10	How much influence has surfing had on your appreciation and respect for ocean ecosystems?	Multi-answer (5 options include other, Likert scale)
11	Have you become aware of plastic pollution in ocean ecosystems through surfing?	Multi-answer (6 options include “open” other)
12	Would you consider any of the following options (at any location you surf)? Leave blank if no.	Multi-answer (6 options)
13	If you can confirm the above points, and would like to join the Focus group study, please leave your email address for the researcher who will get back to you before 1 July 2020. Participants are under no obligation to commit when registering interest and only a small selection will be needed for the study.	Email address

## Appendix B

**Table A2:** Demographics of chosen participants. The highest frequencies of surfer variables found in the survey results are in bold.

Particpant	Gender	Age Range	Country from	County in	Local Break	4× Surfer Variables			
						Experience	Frequency	Localisation	Ability
A	Female	35–44	Wales	Wales	Aberystwyth Trap/Borth	**Over 10 years**	Once or twice a month	**Moves between a few local spots**	**Intermediate**
B	Female	26–34	England	Portugal	Tonel	2–5 years	**1–5 times a week**	**Moves between a few local spots**	**Intermediate**
C	Male	45–54	South Africa	England	Croyde	**Over 10 years**	**1–5 times a week**	Always local break	**Intermediate**
D	Female	26–34	Wales	Wales	Manobier/Freshwater West	5–10 years	Once or twice a month	**Moves between a few local spots**	Improver
E	Female	35–44	England	England	Crode/Saunton	5–10 years	Once or twice every 6 months	Only holidays or weekends	Improver
F	Male	18–25	Italy	France	Quiberon/Côte Sauvage (French Britanny)	1–2 years	Every day	Surfs local break and travels extensively	Improver

## Appendix C

Email sent to participants chosen for the focus group.

Please think about your relationship to surfing and the ocean.

Please read the following statement and consider if it is the same for you.

“I sometimes feel that riding waves is all about reading waves. Surfing requires ocean literacy, a knowledge and understanding of the swell, currents and wind that allows the surfer to navigate the sea and to create something beautiful with the waves" Ava Parsemain. With this quote as a starting point, we will discuss each participants perspective on their relationship between surfing and ocean awareness. Each participant will be given a letter A-F and we will go in alphabetical order. We will focus on 3 sections to help tell a concise narrative of this relationship including the following.

Benefits; things you receive and appreciate about surfing.

Risks; has there been negative impacts/affects/experiences.

Barriers; what has stopped/hindered you—or other people—from going surfing?

Ocean Literacy definition:

Ocean literacy is an understanding of the ocean’s influence on you—and your influence on the ocean. An ocean-literate person understands the essential principles and fundamental concepts about the functioning of the ocean. They can communicate about the ocean in a meaningful way and are able to make informed and responsible decisions regarding the ocean and its resources.

## Appendix D

These are the 6 emergent themes from the coded focus group transcript with identified Parent themes. Parent themes were assigned by identifying the appropriate category in the Surfing Social Ecological System (Resource user, Governance, Resource unit and Resources Systems)

- Conditions, variables and awareness of interconnections (parent theme: SES resource system)
- Plastic pollution evidence and barriers (parent theme: SES user)
- Personal motivations to surf; positive impacts of surfing (parent theme: SES user)
- Negative impacts of microplastic pollution (parent theme: SES user)
- Self motivation to learn about ocean sustainability issues (parent theme: SES user)
- Origins of understanding ocean sustainability issues (parent theme: SES user)

## Appendix E

**Table A3:** This is a contingency table displaying data from surfer variables (questions 1–4) and Ocean Literacy Principles (question 8).

		Experience					Frequency					Localisation					Ability				
		A	B	C	D	** E*	F	G	H	** I*	J	K	L	M	** N*	O	P	Q	** R*	S	T
OL1	* **Yes**	11	13	39	35	110	15	32	48	97	11	27	22	33	92	32	21	48	93	45	3
	No	2	2	13	9	22	0	8	18	18	2	6	6	6	23	5	3	13	20	12	0
OL2	* **Yes**	11	12	43	39	117	**10**	**35**	**54**	**107**	**11**	33	22	27	92	32	20	50	101	49	3
	No	0	2	4	6	10	**4**	**5**	**5**	**5**	**3**	6	6	6	23	5	1	7	9	5	0
OL3	*** Yes**	12	13	46	40	125	15	37	57	109	13	38	28	32	102	33	22	53	106	54	3
	No	0	0	2	2	4	0	1	2	5	0	0	1	1	1	1	0	2	5	1	0
OL4	*** Yes**	12	12	41	36	118	13	35	51	105	9	34	25	31	95	31	20	51	99	47	3
	No	0	1	6	6	9	2	2	8	7	3	3	1	3	10	4	2	5	9	6	0
OL5	*** Yes**	12	13	47	43	130	15	38	59	115	13	38	29	33	108	34	22	55	115	55	3
	No	0	0	0	0	0	0	0	0	0	0	0	0	0	0	0	0	0	0	0	0
OL6	*** Yes**	12	13	44	41	122	**15**	**37**	**54**	**109**	**12**	36	28	30	101	34	22	53	105	51	3
	No	0	0	4	1	8	**1**	**6**	**4**	**1**	**1**	3	0	3	7	0	1	2	6	4	0
OL7	*** Yes**	12	12	44	38	121	15	35	54	107	12	35	25	33	102	30	22	52	100	52	3
	No	0	1	1	6	7	3	3	3	10	1	6	1	2	7	4	2	3	13	3	0

The answers across the rows and columns with highest frequencies have a * and are in bold. Ocean Literacy Principle 5 was except from calculations due to too many zeros. The chi-squared tests for independence showed Frequency x OL2 *p*-value = 0.01899 and Frequency x OL6 *p*-value = 0.03348. These values are in the table in bold. With *p*-values < 0.05 these are the only data with statistical significance, where we can reject the null hypothesis of independence.

## References

[1] Worm B., Barbier E.B., Beaumont N., Duffy J.E., Folke C., Halpern B.S., Jackson J.B., Lotze H.K., Micheli F., Palumbi S.R. (2006). Impacts of biodiversity loss on ocean ecosystem services. Science.

[2] Bijma J., Pörtner H., Yesson C., Rogers A.D. (2013). Climate change and the oceans—What does the future hold?. Mar. Pollut. Bull..

[3] Cózar A., Echevarría F., González-Gordillo J.I., Irigoien X., Úbeda B., Hernández-León S., Palma Á.T., Navarro S., García-de-Lomas J., Ruiz A. (2014). Plastic debris in the open ocean. Proc. Natl. Acad. Sci. USA.

[4] Gall S.C., Thompson R.C. (2015). The impact of debris on marine life. Mar. Pollut. Bull..

[5] Rudolph T.B., Ruckelshaus M., Swilling M., Allison E.H., Österblom H., Gelcich S., Mbatha P. (2020). A transition to sustainable ocean governance. Nat. Commun..

[6] Halpern B.S., Frazier M., Afflerbach J., Lowndes J.S., Micheli F., O’Hara C., Scarborough C., Selkoe K.A. (2019). Recent pace of change in human impact on the world’s ocean. Sci. Rep..

[7] Duarte C.M., Agusti S., Barbier E., Britten G.L., Castilla J.C., Gattuso J., Fulweiler R.W., Hughes T.P., Knowlton N., Lovelock C.E. (2020). Rebuilding marine life. Nature.

[8] Ryabinin V., Barbière J., Haugan P., Kullenberg G., Smith N., McLean C., Troisi A., Fischer A., Aricò S., Aarup T. (2019). The UN decade of ocean science for sustainable development. Front. Mar. Sci..

[9] Dupont S., Fauville G., Nunes P.A.L.D., Svensson L.E., Markandya A. (2017). Ocean literacy as a key toward sustainable development and ocean governance. Handbook on the Economics and Management of Sustainable Oceans.

[10] Steel B.S., Smith C., Opsommer L., Curiel S., Warner-Steel R. (2005). Public ocean literacy in the United States. Ocean Coast. Manag..

[11] Kelly R., Evans K., Alexander K., Bettiol S., Corney S., Cullen-Knox C., Cvitanovic C., de Salas K., Emad G.R., Fullbrook L. (2021). Connecting to the oceans: Supporting ocean literacy and public engagement. Rev. Fish Biol. Fish..

[12] Santoro F., Selvaggia S., Scowcroft G., Fauville G., Tuddenham P. (2017). Ocean Literacy for All: A Toolkit.

[13] IOC, UNESCO Ocean Knowledge for a Sustainable Ocean Economy: Synergies between the Ocean Decade and the Outcomes of the Ocean Panel. https://oceandecade.com/assets/uploads/documents/Ocean-Panel-Ocean-Decade-FINAL-compressed_1616142993.pdf.

[14] Brennan C., Ashley M., Molloy O. (2019). A system dynamics approach to increasing ocean literacy. Front. Mar. Sci..

[15] Britton E., Domegan C., McHugh P. (2021). Accelerating sustainable ocean policy: The dynamics of multiple stakeholder priorities and actions for oceans and human health. Mar. Policy.

[16] Fleming L., Depledge M., McDonough N., White M., Pahl S., Austen M., Goksoyr A., Solo-Gabriele H., Stegeman J. (2015). The oceans and human health. Oxf. Res. Encycl. Environ. Sci..

[17] Fleming L.E., Bruce M., Mathew P.W., Michael H.D. (2019). Fostering human health through ocean sustainability in the 21st century. People Nat..

[18] Seas Oceans and Humans Health in Europe. https://sophie2020.eu/strategic-research-agenda/target-area-2/.

[19] Völker S., Heiler A., Pollmann T., Claßen T., Hornberg C., Kistemann T. (2018). Do perceived walking distance to and use of urban blue spaces affect self-reported physical and mental health?. Urban For. Urban Green..

[20] Bratman G.N., Anderson C.B., Berman M.G., Cochran B., De Vries S., Flanders J., Folke C., Frumkin H., Gross J.J., Hartig T. (2019). Nature and mental health: An ecosystem service perspective. Sci. Adv..

[21] Martin L., White M.P., Hunt A., Richardson M., Pahl S., Burt J. (2020). Nature contact, nature connectedness and associations with health, wellbeing and pro-environmental behaviours. J. Environ. Psychol..

[22] Gascon M., Zijlema W., Vert C., White M.P., Nieuwenhuijsen M.J. (2017). Outdoor blue spaces, human health and well-being: A systematic review of quantitative studies. Int. J. Hyg. Environ. Health.

[23] Corne N.P. (2009). The implications of coastal protection and development on surfing. J. Coast. Res..

[24] Lazarow N. (2007). The value of coastal recreational resources: A case study approach to examine the value of recreational surfing to specific locales. J. Coast. Res..

[25] Ponting J., O’Brien D. (2014). Liberalizing Nirvana: An analysis of the consequences of common pool resource deregulation for the sustainability of Fiji’s surf tourism industry. J. Sustain. Tour..

[26] Scarfe B.E., Healy T.R., Rennie H.G., Mead S.T. (2009). Sustainable management of surfing breaks: Case studies and recommendations. J. Coast. Res..

[27] Anderson J. (2014). Surfing between the local and the global: Identifying spatial divisions in surfing practice. Trans. Inst. Br. Geogr..

[28] Lewin J., Schaefer C.T., McLachlan A., Erasmus T. The Role of Phytoplankton in Surf Ecosystems. Sandy Beaches as Ecosystems.

[29] Borne G. (2018). Surfing and Sustainability.

[30] Lazarow N., Olive R. (2017). Culture, meaning and sustainability in surfing. Sustainable Surfing.

[31] Arroyo M., Levine A., Espejel I. (2019). A transdisciplinary framework proposal for surf break conservation and management: Bahía de Todos Santos World Surfing Reserve. Ocean Coast. Manag..

[32] Arroyo M., Levine A., Brenner L., Seingier G., Leyva C., Espejel I. (2020). Indicators to measure pressure, state, impact and responses of surf breaks: The case of Bahía de Todos Santos World Surfing Reserve. Ocean Coast. Manag..

[33] Martin S.A., Assenov I. (2014). Investigating the importance of surf resource sustainability indicators: Stakeholder perspectives for surf tourism planning and development. Tour. Plan. Dev..

[34] Larson L.R., Usher L.E., Chapmon T. (2018). Surfers as environmental stewards: Understanding place-protecting behavior at Cape Hatteras National Seashore. Leis. Sci..

[35] Scheske C., Arroyo Rodriguez M., Buttazzoni J.E., Strong-Cvetich N., Gelcich S., Monteferri B., Rodríguez L.F., Ruiz M. (2019). Surfing and marine conservation: Exploring surf-break protection as IUCN protected area categories and other effective area-based conservation measures. Aquat. Conserv..

[36] Atkin E., Bryan K., Mead S., Hume T., Waiti J. (2019). Management Guidelines for Surfing Resources. Proceedings of the Australasian Coasts and Ports 2019 Conference: Future Directions from 40 [Degrees] S and Beyond.

[37] Ingersoll K.A. (2016). Waves of Knowing: A Seascape Epistemology.

[38] Mills J., Bahfen N. (2014). Changing patterns and critical dialogues: New uses of literacy. Fusion J..

[39] Reineman D.R. (2016). The utility of surfers’ wave knowledge for coastal management. Mar. Policy.

[40] Hutt J.A., Black K.P., Mead S.T. (2001). Classification of surf breaks in relation to surfing skill. J. Coast. Res..

[41] Armitano C.N., Clapham E.D., Lamont L.S., Audette J.G. (2015). Benefits of surfing for children with disabilities: A pilot study.

[42] Caddick N., Smith B., Phoenix C. (2015). The effects of surfing and the natural environment on the well-being of combat veterans. Qual. Health Res..

[43] Hignett A., White M.P., Pahl S., Jenkin R., Froy M.L. (2018). Evaluation of a surfing programme designed to increase personal well-being and connectedness to the natural environment among ‘at risk’young people. J. Adventure Educ. Outdoor Learn..

[44] Matos M.G., Santos A.C., Fauvelet C., Marta F., Evangelista E.S., Ferreira J., Moita M., Conibear T., Mattila M. (2017). Surfing for social integration: Mental health and well-being promotion through surf therapy among institutionalized young people. J. Community Med. Public Health Care.

[45] Creswell J.W. (2017). Research Design: Qualitative, Quantitative, and Mixed Methods Approaches.

[46] Britton E., Foley R. (2020). Sensing water: Uncovering health and well-being in the sea and surf. J. Sport Soc. Issues.

[47] Online Surveys Surfing Survey Page. https://angliaruskin.onlinesurveys.ac.uk/surfingsurvey.

[48] Fox N. Eco Yoga Surf Home Page. https://www.ecoyogasurf.com.

[49] Noy C. (2008). Sampling knowledge: The hermeneutics of snowball sampling in qualitative research. Int. J. Soc. Res. Methodol..

[50] Hermoso M., Narváez S., Thiel M. (2021). Engaging recreational scuba divers in marine citizen science: Differences according to popularity of the diving area. Aquat. Conserv. Mar. Freshw. Ecosyst..

[51] Bernard H.R. (2013). Social Research Methods: Qualitative and Quantitative Approaches.

[52] Joshi A., Kale S., Chandel S., Pal D.K. (2015). Likert Scale: Explored and Explained. Curr. J. Appl. Sci. Technol..

[53] Costa S., Caldeira R. (2018). Bibliometric analysis of ocean literacy: An underrated term in the scientific literature. Mar. Policy.

[54] Usher L.E., Goff J., Gómez E. (2016). Exploring surfers’ perceptions of municipal regulations using grounded theory. Ann. Leis. Res..

[55] Tunstall S.M., Penning-Rowsell E.C. (1998). The English beach: Experiences and values. Geogr. J..

[56] Ostrom E. (2009). A general framework for analyzing sustainability of social-ecological systems. Science.

[57] Leslie H.M., Basurto X., Nenadovic M., Sievanen L., Cavanaugh K.C., Cota-Nieto J.J., Erisman B.E., Finkbeiner E., Hinojosa-Arango G., Moreno-Báez M. (2015). Operationalizing the social-ecological systems framework to assess sustainability. Proc. Natl. Acad. Sci. USA.

[58] Schlüter M., Hinkel J., Bots P.W., Arlinghaus R. (2014). Application of the SES framework for model-based analysis of the dynamics of social-ecological systems. Ecol. Soc..

[59] McGinnis M.D., Ostrom E. (2014). Social-ecological system framework: Initial changes and continuing challenges. Ecol. Soc..

[60] Fauville G., Strang C., Cannady M.A., Chen Y. (2019). Development of the International Ocean Literacy Survey: Measuring knowledge across the world. Environ. Educ. Res..

[61] Lin Y.-L., Wu L.-Y., Tsai L.-T., Chang C.-C. (2020). The Beginning of Marine Sustainability: Preliminary Results of Measuring Students’ Marine Knowledge and Ocean Literacy. Sustainability.

[62] Reineman D.R., Ardoin N.M. (2018). Sustainable tourism and the management of nearshore coastal places: Place attachment and disruption to surf-spots. J. Sustain. Tour..

[63] Ferreira J.C., Vasconcelos L., Monteiro R., Silva F.Z., Duarte C.M., Ferreira F. (2021). Ocean literacy to promote sustainable development goals and agenda 2030 in coastal communities. Educ. Sci..

[64] De Bell S., Graham H., Jarvis S., White P. (2017). The importance of nature in mediating social and psychological benefits associated with visits to freshwater blue space. Landsc. Urban Plan..

[65] Viatori M., Scheuring B. (2020). Saving the costa verde’s waves: Surfing and discourses of race–class in the enactment of lima’s coastal infrastructure. J. Lat. Am. Caribb. Anthropol..

[66] Ponting J., O’Brien D. (2015). Regulating “Nirvana”: Sustainable surf tourism in a climate of increasing regulation. Sport Manag. Rev..

[67] Marshall J., Ferrier B., Ward P.B., Martindale R. (2020). “I feel happy when I surf because it takes stress from my mind”: An Initial Exploration of Program Theory within Waves for Change Surf Therapy in Post-Conflict Liberia. J. Sport Dev..

[68] Armstrong C. (2020). Ocean justice: SDG 14 and beyond. J. Glob. Ethics.

[69] Mishra H.S., Bell S., Vassiljev P., Kuhlmann F., Niin G., Grellier J. (2020). The development of a tool for assessing the environmental qualities of urban blue spaces. Urban For. Urban Green..

[70] Britton E., Kindermann G., Domegan C., Carlin C. (2020). Blue care: A systematic review of blue space interventions for health and wellbeing. Health Promot. Int..

[71] Drake C.J., Keith M., Dober M.R., Evans S., Olive L.S. (2021). A qualitative investigation into the perceived therapeutic benefits and barriers of a surf therapy intervention for youth mental health. Complementary Ther. Med..

[72] Britton E., Kindermann G., Carlin C. (2020). Surfing and the sense: Using body mapping to understand the embodied and therapeutic experiences of young surfers with Autism. Glob. J. Community Psychol. Pract..

[73] Olive R., Wheaton B. (2021). Understanding blue spaces: Sport, bodies, wellbeing, and the sea. J. Sport Soc. Issues.

[74] Wheaton B., Waiti J.T.A., Olive R., Kearns R. (2021). Coastal communities, leisure and wellbeing: Advancing a trans-disciplinary agenda for understanding ocean-human relationships in aotearoa New Zealand. Int. J. Environ. Res. Public Health.

[75] Ruttenberg T., Brosius P. (2017). Decolonizing Sustainable Surf Tourism. The Critical Surf Studies Reader.

[76] Pearlman J., Bushnell M., Coppola L., Karstensen J., Buttigieg P.L., Pearlman F., Simpson P., Barbier M., Muller-Karger F.E., Munoz-Mas C. (2019). Evolving and sustaining ocean best practices and standards for the next decade. Front. Mar. Sci..

[77] Sacco K., Falk J.H., Bell J. (2014). Informal science education: Lifelong, life-wide, life-deep. PLoS Biol..

[78] Meadows D.H., Meadows D.L., Randers J., Behrens W.W. (1972). The Limits to Growth.

[79] Hardin G. (1968). The tragedy of the commons. Science.

[80] Nazer D. (2004). The tragicomedy of the surfers’ commons. Deakin Law Rev..

[81] Doering A., Higham J., Hinch T. (2018). From he’e nalu to olympic sport: A century of surfing evolution. Sport Tourism Development.

[82] Ashley M., Pahl S., Glegg G., Fletcher S. (2019). A change of mind: Applying social and behavioral research methods to the assessment of the effectiveness of ocean literacy initiatives. Front. Mar. Sci..

[83] Brewin R.J., de Mora L., Jackson T., Brewin T.G., Shutler J. (2015). On the potential of surfers to monitor environmental indicators in the coastal zone. PLoS ONE.

[84] Leonard A.F., Garside R., Ukoumunne O.C., Gaze W.H. (2020). A cross-sectional study on the prevalence of illness in coastal bathers compared to non-bathers in England and Wales: Findings from the beach user health survey. Water Res..

[85] Kapono C. (2018). A Multi-Cultural Characterization of Human-Environmental Interaction through Metabolomic and Microbiome Profiling. https://escholarship.org/uc/item/4t04z4rp.

[86] Scott V. (2019). Who will Surf for Science? Understanding Motivations to Engage Surfers in Citizen Science with Smartfin. https://escholarship.org/uc/item/4qc3z161.

[87] Brewin R.J., Cyronak T., Bresnahan P.J., Andersson A.J., Richard J., Hammond K., Billson O., de Mora L., Jackson T., Smale D. (2020). Comparison of two methods for measuring sea surface temperature when surfing. Multidiscip. Digit. Publ. Inst..

[88] Weber A.K., Weber M.W., Savoca M.S. (2019). Quantifying marine debris associated with coastal golf courses. Mar. Pollut. Bull..

[89] Camins E., de Haan W.P., Salvo V., Canals M., Raffard A., Sanchez-Vidal A. (2020). Paddle surfing for science on microplastic pollution. Sci. Total Environ..

[90] Whyte D. (2019). Belonging in the Ocean: Surfing, ocean power, and saltwater citizenship in Ireland. Anthropol. Noteb..

[91] Visbeck M. (2018). Ocean science research is key for a sustainable future. Nat. Commun..

[92] Anderson J., Peters K. (2014). A perfect and absolute blank’: Human geographies of water worlds. Water Worlds: Human Geographies of the Ocean.

[93] George R.Y., Wiebe S.M. (2020). Fluid Decolonial Futures: Water as a Life, Ocean Citizenship and Seascape Relationality. New Political Sci..

[94] Waitt G. (2008). ‘Killing waves’: Surfing, space and gender. Soc. Cult. Geogr..

[95] McCauley V., McHugh P., Davison K., Domegan C. (2019). Collective intelligence for advancing ocean literacy. Environ. Educ. Res..

[96] Fletcher S., Potts J. (2007). Ocean citizenship: An emergent geographical concept. Coast. Manag..

[97] Stoll-Kleemann S. (2019). Feasible options for behavior change toward more effective ocean literacy: A systematic review. Front. Mar. Sci..

[98] Fleming L.E., Depledge M., Bouley T., Britton E., Dupont S., Eatock C., Garside R., Heymans J.J., Kellett P., Lloret J. (2021). The ocean decade—Opportunities for oceans and human health programs to contribute to public health. Am. J. Public Health.

[99] Polejack A. (2021). The Importance of Ocean Science Diplomacy for Ocean Affairs, Global Sustainability, and the UNDecade of Ocean Science. Front. Mar. Sci..

[100] Claudet J., Bopp L., Cheung W.W., Devillers R., Escobar-Briones E., Haugan P., Heymans J.J., Masson-Delmotte V., Matz-Lück N., Miloslavich P. (2020). A roadmap for using the UN decade of ocean science for sustainable development in support of science, policy, and action. One Earth.

